# The modulation of sustainability knowledge and impulsivity traits on the consumption of foods of animal and plant origin in Italy and Turkey

**DOI:** 10.1038/s41598-022-24325-z

**Published:** 2022-11-21

**Authors:** Riccardo Migliavada, Carol Coricelli, Esra Emine Bolat, Ceyhun Uçuk, Luisa Torri

**Affiliations:** 1 University of Gastronomic Sciences, Piazza Vittorio Emanuele II 9, 12042 Pollenzo, Italy; 2 Western University, 1151 Richmond St, London, ON N6A 3K7 Canada; 3 Gaziantep University, Üniversite Blv., 27310 Şehitkamil-Gaziantep, Turkey

**Keywords:** Psychology, Human behaviour

## Abstract

Given the environmental challenge we face globally, a transition to sustainable diets seems essential. However, the cognitive aspects underlying sustainable food consumption have received little attention to date. The aims of this cross-cultural study were: (1) to explore how impulsivity traits and individuals’ knowledge of food environmental impact influence their frequency of consumption of animal- and plant-based foods; (2) to understand the modulation of individual characteristics (i.e. generation, sex, BMI, and sustainability knowledge). An online survey investigating impulsivity traits, sustainability knowledge and ratings of diverse food items was designed and administered to respondents from Italy (N = 992) and Turkey (N = 896). Results showed that Turkish respondents were higher in impulsivity and animal products consumption. Italians, instead, had greater sustainability knowledge and consumed more plant-based foods. Females in both groups reported greater knowledge of sustainability, consistent with previous findings. In terms of generations, the lowest consumption of animal products was reported by Turkish Generation Z and Italian Millennials. In conclusion, this study shed light on the interaction of psychological factors and individual characteristics with the perceived environmental impact of foods. Moreover, the adopted cross-cultural approach allowed to identify several differences in participants’ responses ascribable to their different nationalities and gastronomic cultures.

## Introduction

The current global environmental challenge requires a transition toward more sustainable diets^1^, namely rich in plant-based products, consuming foods with a low carbon footprint and recycling of edible food waste^2^. However, governments, industry, and civil society organizations are mostly focused on reducing food loss and waste, and promoting technological innovations and new business strategies, while underestimating the relative contribution of dietary change to sustainable food systems, even though it has the potential to be an effective approach^3^. To change eating habits and diets, however, individuals need a general knowledge of food environmental impact, but also a radical transformation of their daily food choices, while, at the same time, policy makers need an understanding of the inherent mechanisms of the human brain in response to food. Yet, food choices are complex and multidimensional, and as such, deeply influenced by psychological and sociocultural factors^4^. This study aims to explore the links between environmental sustainability knowledge, psychological traits, sociocultural differences and eating behaviors.

Food is a multiattribute stimulus and each time it is encountered in the environment, in order to make a choice, its value has to be assessed^5^. Such evaluations rely on the attributes that determine the food (e.g., taste, caloric content), the current psycho-physiological state of the individual (i.e., hunger level) and the memories (pleasure vs. disgust or nausea) associated with eating such food in the past, but also on more abstract attributes looking at the future wellbeing of the individual (i.e. healthiness)^5^. Since different evaluations may coexist for a given food (e.g., holding an automatic implicit positive evaluation and an explicit negative evaluation for French fries^6,7^), previous literature has proposed the existence of two separate systems, one reflective and one impulsive, which produce different behavioral outcomes depending on whether the decision is based on knowledge and values (i.e., healthiness or other abstract attributes) or on motivational (rewarding) orientations (based on taste or palatability)^6^. Among abstract attributes, environmental sustainability has received increasing attention in recent years^8^, but so far, no study has explored how impulsivity traits and knowledge of the environmental impact of food influence the frequency of consumption of animal and plant-based foods.

Impulsivity, which is widely acknowledged as a complex multifaceted construct, is one of the most relevant psychological traits modulating food choices^9^. Traits of impulsivity include the inability to plan ahead, attentive difficulties, and thoughtless and unplanned reactions to internal or external stimuli without consideration of negative consequences^10,11^. There has been some debate regarding the relationship between impulsivity and reward sensitivity, and how independent these two constructs are^12,13^. Nevertheless, using the well-established Go/No-go task^14^, overweight and obese individuals showed greater difficulty in inhibiting responses to foods^15^. Fewer studies have addressed the role of food-related impulsivity in normal weight healthy subjects. Indeed, most studies focused on external eating (i.e., an increased tendency to eat in response to external cues, such as the sight or smell of food) and its relationship with impulsivity and attentional bias to food cues^16^. Hare and colleagues^17^ reported that, in a forced choice task, normal weight healthy subjects with low impulsivity and high self-control (self-controllers) were able to refuse to choose items previously rated as high in tastefulness but low in healthiness, whereas non-self-controllers made decisions based on taste alone. Impulsivity traits have been positively correlated with present hedonism (i.e., the tendency of some individuals to prefer immediate pleasure over future rewards) and overeating (i.e. obese individuals with higher impulsivity traits have shown less inhibitory control in food-related tasks^18,19^), and negatively correlated with future orientation^9^. Since opting for a sustainable diet and reducing the consumption of animal-based foods means to be aware of the long-term consequences of both one’ s personal health and the future of the planet, impulsivity traits may play an important part. However, what is the impact of psychological traits (i.e. impulsivity) and other individual characteristics (i.e. generation, sex, Body Mass Index/BMI) on sustainable food choices has yet to be fully clarified.

Although a shift toward sustainable diets is urgent, little is known about individuals’ evaluations of foods with respect to this abstract and future driven dimension. Are consumers aware of the environmental impact of their diets? Do consumers acknowledge differences in the greenhouse gas (GHG) emissions of specific foods? Preliminary results showed that when explicitly asked to self-report their sustainable food consumption participants reported a high level of sustainability choices^20^. Nevertheless, these results might be influenced by the social desirability bias in which participants tend to under-report socially undesirable behaviors included in the food domain^7^.

In the attempt to answer the question whether individual characteristics, such as sex or generation, influence sustainability knowledge and awareness, early studies have shown that men tend to report a stronger preference towards meat than women^21^, while the latter are more likely to adopt a vegetarian lifestyle^22^. More recently, males were found to view a meal as incomplete if lacking meat^23^ and cultural associations between consumption of meat and so-called “masculinity” have been reported^24,25^. Age, education and socioeconomic status have also been linked to vegetable consumption, with low consumption of vegetables being associated with lower education and socioeconomic status^26^, and younger age^27^. Sociodemographic characteristic such as sex, age and education were all found to affect sustainability knowledge and pro-environmental behaviors^28,29^, however, since human–environment interactions are culture-bound, these differences are not just at the individual level^30^.

Cultural factors, in fact, modulate food evaluations and preferences throughout lifespan; countries’ traditions shape individuals’ taste perception and the frequency they are exposed to certain flavors^31–33^; they influence what foods people eat and what they do not eat (i.e. food taboos and religious food restrictions^34^). Finally, living in an urbanized city or a rural area affects which foods one is exposed to and the recipes one includes in their daily diet^35^. Another reason why it is important to consider cultural influences on environmental attitudes and behaviors^36^ is, for example, the fact that some societies have shown weaker association between environmental concern and pro-environmental behavior than others^37^. Even the notion that women are generally more pro-environment than men has recently been linked to sociocultural contexts. In fact, according to Chan and colleagues^38^, gender differences in environmental concern are indeed smaller in societies with higher levels of gender inequality, economic scarcity, power distance, and collectivism. To date, only few studies^39–41^ have directly addressed the impact of sustainability knowledge and psychological traits of individuals on food choices by also considering cultural factors. Given the observed lack of studies, the present study seeks to fill this gap.

The aim of this study was threefold. Firstly, we investigated the role of impulsivity traits and participants’ knowledge of the environmental impact of food in influencing the frequency of consumption of animal- and plant-based foods. Despite the exploratory nature of the present study, we hypothesized that participants with higher levels of impulsivity traits and a lower sustainability knowledge of animal-based foods would have higher levels of animal-based foods consumption compared to participants with lower impulsivity traits and higher sustainability knowledge of animal-based foods. Thus, we hypothesize that an interaction may exist between impulsivity and knowledge of the sustainability of a food, and that this could affect the consumption of that food. Secondly, we wanted to understand how sociodemographic characteristics (including sex, level of education and generation), BMI, food preferences and sustainability knowledge (assessed through a questionnaire) of participants modulate the evaluation and the consumption of both animal- and plant-based food products. Based on previous literature, we hypothesized to find sex differences in our results with females consuming less meat and having a more sustainable lifestyle including the food domain^24,25,42^. Thirdly, in a cross-cultural comparison perspective we wanted to investigate whether there were differences between Italian and Turkish participants, in a first-of-a-kind comparison on this topic between two populations. These two countries were chosen because, despite their relative geographical proximity and a common Mediterranean dietary pattern, there are strong differences in environmental politics and sociocultural differences including economical and religious differences, which are known to affect food taboos and choices^43^.

## Materials and methods

### Online survey

An online self-reported survey composed of 56 questions was designed using the software Qualtrics^®^ (Qualtrics, Provo, UT). The survey was developed in English and back translated into Italian (ITA) and Turkish (TUR), and was then distributed using a link emailed to potential respondents and disseminated through social media (e.g., LinkedIn, WhatsApp, Facebook). Data were collected anonymously between April and September 2021. The online study consisted of four separate sections with the following order: (i) sociodemographic characteristics and eating habits questionnaire; (ii) explicit ratings on food liking, frequency of consumption and perceived environmental impact of animal- and plant-based foods; (iii) impulsivity assessment questionnaire; (iv) sustainability knowledge assessment questionnaire.

#### Sociodemographic characteristics and eating habits

In the first section, all participants were asked to provide sociodemographic, anthropometric and eating habits data including: sex, age, body weight (kg), height (cm), nationality, level of education (Primary school, Middle school, Some High School, High school graduate, Bachelor’s Degree, Master’s Degree, Ph.D.), type of diet (Vegan, Vegetarian, Flexitarian, Omnivorous), dietary restrictions (None, Allergies or Intolerances, Religious beliefs, Other personal reasons), and location of residence (City, Suburbs, Countryside).

#### Liking, frequency of consumption and environmental impact of food items

In the second section, participants had to rate food items according to their stated liking, frequency of consumption and perceived environmental impact. Food items were selected based on data by Poore and Nemecek^44^ reporting GHG values for animal- and plant-based foods. The comprehensive database of foods includes multiple impacts based on five environmental indicators. We selected five *animal* products (poultry meat, bovine meat, farmed fish, eggs, cheese) and five *plant-based* products (vegetables, pulses, tofu, nuts, fruits). Participants rated each of the 10 food items answering three questions related to three dimensions (in brackets the two labels at the extremes of the Likert-type scale):

- Liking: “How much do you like this food?” (1 = “Dislike very much” and 7 = “Like very much”).
- Frequency of consumption: “How often do you eat this food” (1 = “Less than once a month” and 5 = “At least once a day”).
- Perceived environmental impact: “How much does the consumption of this food negatively impact the planet?” (1 = “Definitely not” and 7 = “Definitely yes”).

The order of food items presentation was randomized across participants.

The above ratings were performed before assessing participants’ sustainability knowledge and impulsivity traits via questionnaires in order to minimize the impact of the social desirability bias.

#### Impulsivity assessment

In the third section, in order to assess the impulsivity traits of the respondents we adopted a shorter version of the Barratt Impulsiveness Scale (BIS-11)^45^. BIS-11 scale, in its many forms, is one of the most widely self-reported impulsivity measures and has been proven to be reliable across several cultures^46^. The original scale consists of 30 questions, which over the years have been grouped into three second-order subscales (i.e., *Attentional*, *Motor*, *Non Planning*) and, lately, in six first-order subscales (i.e., *Attention*, *Motor*, *Self-control*, *Cognitive complexity*, *Perseverance*, *Cognitive instability*) in an attempt to measure the different components of impulsivity and to better express its multi-faceted nature^46^. Several shorter versions of the scale were also proposed by various authors^47–49^. The reason we decided to adopt this scale is that, despite some discrepancies, BIS-11, in either its long and short versions, has shown generally acceptable or good indices of validity and reliability. For the purpose of our study, we decided to use the domains *Non-planning impulsivity* (i.e., *Self-control* and *Cognitive complexity*) and *Perseverance* since we expected these subscales to influence impulsivity in the food domain compared to the subscales involving *Motor impulsivity*, therefore excluded here. Our version of the BIS-11 was therefore composed of 15 questions as follows: (i) six from *Self control* sub-domain; (ii) five from *Cognitive complexity*; (iii) four from *Perseverance* domain. Each question was scored on a four-point Likert-type scale (rarely/never, occasionally, often, almost always/always). Nine questions were inversely scored. Individuals with higher scores are those considered more impulsive. Because many attempts to validate BIS-11 have resulted in factor structures that differ from the original or with subscales showing inadequate levels of internal consistency^47^, we decided to report only the total score, as the majority of studies in the literature did. For the Italian version of the scale, we referred to the work of Fossati et al.^10^, while for the Turkish version we used the one developed by Güleç et al.^50^.

#### Sustainability knowledge assessment

The fourth and last section consisted of a questionnaire about food and the environment developed from a cross-cultural study ran by Sánchez-Bravo et al.^51^ on 3600 consumers in six different countries (i.e., Brazil, China, India, Mexico, Spain and USA). Their work aimed at comparing differences in the understanding of the sustainability concept in agricultural products across countries and was organized in two topics: one about general sustainability and the other on the willingness to pay for different food categories. In order to create an index to measure sustainability knowledge, we focused on four of the six domains belonging to the first topic: *basic statements or definitions*, *benefits for local communities*, *price and purchase intention*, *relevance to consumers*. Questions belonging to the other domains were considered irrelevant for our study and discarded. The 22 questions selected were rated on a seven-point Likert-type scale (1 = Strongly disagree; 7 = Strongly agree) and the scores of the questions Q2.3 (Consuming products made from environmentally friendly grains is more expensive than consuming conventional products), Q2.5 (Conventional and highly automated farming leads to higher quality products), Q2.28 (The volume of water needed to grow 1 lb of tomatoes is approximately the same as the amount needed to grow 1 lb of wheat), and Q2.29 (World food production cannot be maintained through local products; intensive agriculture is needed) were reversed. Finally, we calculated the total score to represent the level of sustainability knowledge, with higher scores standing for a greater understanding of sustainability.

### Participants

A total of 2326 responses were collected, of which 221 were excluded since the sections assessing impulsivity and/or sustainability knowledge were not complete and 211 were discarded because they did not meet the inclusion criteria (i.e. out of range age or BMI, not Italian nor Turkish nationality). In particular: 98 were excluded because it was not possible to determine in which of the two countries (Italy or Turkey) the respondents who filled in “other nationalities” lived; 67 were not considered because the subjects were under the age of 18, therefore ineligible to sign informed consent and respond to the survey; 34 were discarded because respondents took less than 5 min to complete the survey, a time that was considered the minimum required to answer all questions; 6 were discarded because they were older than 75 years and outside our inclusion criteria (18–75), as this category of people is more likely to have particular age-related eating habits and is less suitable for online surveys conducted on technological devices (compared to paper-and-pencil surveys and oral interviews); 6 were removed because of the extreme BMI of the respondents out of the range of our inclusion criteria (very severely underweight (i.e. BMI < 15) and very severely obese (i.e. BMI > 50)); 6 were dropped because they were identified as outliers due to inconsistencies in data entry (i.e. subjects systematically reported the lowest values for food liking and the highest values for associated frequency of consumption). The final sample consisted of 1888 respondents: 992 from Italy (M = 38 years old, SD = 13.6, 62.7% females) and 896 from Turkey (M = 26 years old, SD = 11.8, 63.7% females). A detailed description of the demographics data is provided in Table 1. The research was approved by the Ethics Committee of the University of Gastronomic Sciences (Ethics Committee Proceeding n. 2021.2). The work was carried out in accordance with the international ethical guidelines for research involving humans established in the Declaration of Helsinki. All subjects provided written informed consent before beginning the survey.

**Table 1:** Characteristics of the participants (N = 992 Italians, N = 896 Turkish).

Characteristic	ITA	TUR
	N (%)	N (%)
Sex (F)	622 (62.7)	571 (63.7)
Age*	41.3 (13.6) [18, 75]	30.6 (11.8) [18, 75]
**Generation**		
Z	140 (14.1)	461 (51.5)
Millennials	389 (39.2)	251 (28)
X	293 (29.5)	154 (17.2)
Boomers	170 (17.1)	30 (3.3)
**Education**		
Primary school	1 (0.1)	4 (0.4)
Middle school	14 (1.4)	10 (1.1)
Some high school	16 (1.6)	28 (3.1)
High school graduate	267 (26.9)	225 (25.1)
Bachelor’s degree	161 (16.2)	441 (49.2)
Masters’s degree	398 (40.1)	143 (16)
Ph.D.	135 (13.6)	45 (5)
**Education level**		
Low	298 (30)	267 (29.8)
High	694 (70)	629 (70.2)
BMI*	23.3 (3.9) [15, 43.9]	24 (4.6) [15.7, 45.4]
Underweight	63 (6.4)	63 (7)
Normal	673 (67.8)	521 (58.1)
Overweight	197 (19.9)	221 (24.7)
Obese	59 (5.9)	91 (10.2)
**Diet**		
Vegan	7 (0.7)	7 (0.8)
Vegetarian	56 (5.6)	13 (1.5)
Flexitarian	181 (18.2)	86 (9.6)
Omnivorous	748 (75.4)	790 (88.2)
**Dietary restrictions**		
None	751 (75.7)	567 (63.3)
Allergies/intolerances	132 (13.3)	70 (7.8)
Religious beliefs	2 (0.2)	163 (18.2)
Other personal reasons	107 (10.8)	96 (10.7)
**Location of residence**		
City	564 (56.9)	814 (90.8)
Suburbs	212 (21.4)	28 (3.1)
Countryside	216 (21.8)	54 (6)

*Mean SD [min, max].

### Statistical analysis

#### Data pre-processing

The statistical software used for all analysis was R (version 4.1.0)^52^. All the analyses were performed using the R basic package *stats* except where expressly stated. Respondents were classified as belonging to different generational groups based on their age: (i) *Generation Z* age under 26; (ii) *Millennials* from 26 to 39; (iii) *Generation X* from 40 to 55; (iv) *Boomers* age above 55^53–55^. From the height and weight of participants we derived the BMI, which was then classified according to the standard categories developed by the World Health Organization^56^ and labeled as *Underweight* (BMI < 18.5), *Normal* (BMI $\ge$ 18.5 and < 25), *Overweight* (BMI $\ge$ 25 and < 30) or *Obese* (BMI $\ge$ 30). Individuals with a bachelor or a superior degree (Master or PhD) were considered as having a *High* level of education while the rest were classified as having a *Low* one.

Respondents who expressed dietary habits that conflicted with the dietary information provided (N = 74) were reclassified based on their eating habits, in accordance with these definitions: (i) vegans are those who not include any food of animal origin in their diet; (ii) vegetarians are those who do not eat any kind of meat but do consume eggs and/or dairy products; (iii) flexitarians are those who have a primarily vegetarian diet but occasionally eat meat or fish; (iv) omnivores include both animal and plant products in their diet. Finally, we computed the average scores obtained by the five animal- and five plant-based products in the domains *Liking*, *Frequency of Consumption*, and *Environmental impact* for each participant (i.e. animal liking, animal consumption, animal environmental impact and plant-based liking, plant-based consumption, plant-based environmental impact) (see Supplementary Materials Fig. S11 for a detailed distribution of food consumption frequency for both countries). Since the used Likert scales had different numbers of points (five or seven) data were standardized using the z-scores transformation.

#### Scales reliability

The internal consistency, which describes the level of correlation between different items of the same questionnaire, was tested using Cronbach’s alpha reliability coefficient separately for the BIS-11 and sustainability knowledge questionnaires. Moreover, we controlled the correlation between each item and the total score from each questionnaire, corrected for item overlap and scale reliability (i.e., r.cor). Since all items should correlate to the total score, for each questionnaire we looked for items that did not correlate with the overall score from the scale and discarded them. To test the reliability of the impulsivity and sustainability knowledge questionnaires we used the *alpha* function of the *psych* package^57^.

##### Impulsiveness scale

An initial analysis of the internal consistency of our version of BIS-11 provided low values for Cronbach’s alpha (0.62 for the Italian version and 0.66 for the Turkish)^58^. From the evaluation of the item-total correlation (r.cor values), it was noticed that, similarly to Coutlee et al.^59^, the found low internal consistency was mainly due to two items of the *perseverance* domain (16 = *I change jobs*; 21 = *I change residences*) that resulted very poorly correlated^60^ with all the others (r.cor equal to 0.00 and 0.02 for items 16 and 21, respectively). Since the meaning of these two items may be different nowadays than they were in the 1960s, when the Barrat Impulsiveness Scale was initially developed^61^, we decided to discard them. Indeed, as pointed out by Coutlee et al.^59^, it is hard to imagine that events such as changing jobs or residence can be considered in the same way as in the past. After removing both items, $\alpha$ values improved to 0.65 and 0.69, respectively for the Italian and the Turkish questionnaires, which are acceptable levels^58^, and in line with the previous literature reporting $\alpha$ values ranging from 0.80 to 0.62^62,63^. Thus, the final scale resulted in 13 items with a total score ranging from 13 to 52. The obtained average impulsivity score was 27.1 (SD = 4.9; range 14–44) for the total sample, 26.8 (SD = 4.8; range 14–44) for the Italian sample and 27.5 (SD = 5.1; range 15–43) for the Turkish one.

##### Sustainability knowledge

Both the Italian and the Turkish versions of the sustainability knowledge questionnaire had high Cronbach’s alpha (0.85 and 0.8 respectively). However, since Cronbach’s alpha can be affected by the scale length in scales with more than 20 items, we examined the item-total correlations^64^ to detect potentially misinterpreted questions. Two items (i.e., Consuming products made from environmentally friendly grains is more expensive than consuming conventional products and The volume of water needed to grow 1 lb of tomatoes is approximately the same as the amount needed to grow 1 lb of wheat) were found to be poorly correlated with the rest of the items (r.cor values 0.04 and 0.01). Since we assumed that these two items could be considered too technical or potentially misinterpreted by participants, we decided to discard both. Removing those two items, the Cronbach’s alpha values increased respectively to 0.87 and 0.84. Sustainability knowledge was therefore measured using 20 items, with total values ranging from 20 to 140. The obtained average score was 109.7 (SD = 14.52; range 32–140) for the total sample, 114.5 (SD = 12.8; range 32–140) for the Italian sample and 104.5 (SD = 14.6; range 33–140) for the Turkish sample.

#### Data analysis

To assess the differences in the mean scores of the questionnaires between respondents we used Welch’s unequal variances t-test, which is more reliable when two samples have unequal variances and/or unequal sample sizes compared to Student’s t-test^65,66^.

To quantify the magnitude of the differences between means (effect size) we computed the Cohen’s d. According to Cohen, d = 0.2 is considered a small effect size, 0.5 represents a medium effect size and 0.8 a large effect size^67^.

We hypothesized that an interaction may exist between impulsivity and participants’ knowledge of the sustainability of a food, and that this could affect the consumption of that food. To investigate these effects, we used multiple linear regression models. Given the hypothesis about the nature of the interaction, the sign of the variable *animal environmental impact* and *plant-based environmental impact* were changed. A first model was created with *animal products consumption* as a dependent variable and *impulsivity*, *animal environmental impact* (negative) and their interaction as independent variables. The models were tested on the Italian and Turkish samples separately to explore the differences between the two populations. Subsequently, we adjusted the model for sociodemographic characteristics (i.e., sex, generation, location, education level), BMI and eating behaviors (i.e., diet, dietary restrictions, food preferences) since they are important factors in influencing food consumption. Finally, since the fact that women are generally more pro-environment than men has been related to sociocultural contexts, we added the interaction effect of sex and knowledge of sustainability as predictor. The same approach was used to exploit the consumption of plant-based foods. For both models the same predictors were used. A complete description of each independent variable, which operationalization was described in previous paragraphs, is provided in Table 1. The Shapiro–Wilk tests^68^ showed that residuals were not normally distributed, except for the Italians’ animal consumption model; however, normal probability plots showed that they were close to normality. The analysis of the residuals of the regression models is reported in the Supplementary Materials (SM) (see Figs. S9, S10). Moreover, given our large sample size, this should not cause major problems in using parametric procedures^69^. According to the central limit theorem, with sample sizes > 30 the sampling distribution of the sample means tends to be normal^70^. Moreover, Gaussian models have been found to be fairly robust to non-normality^71^.

## Results

### Main differences between Italian and Turkish populations

**Figure 1:**
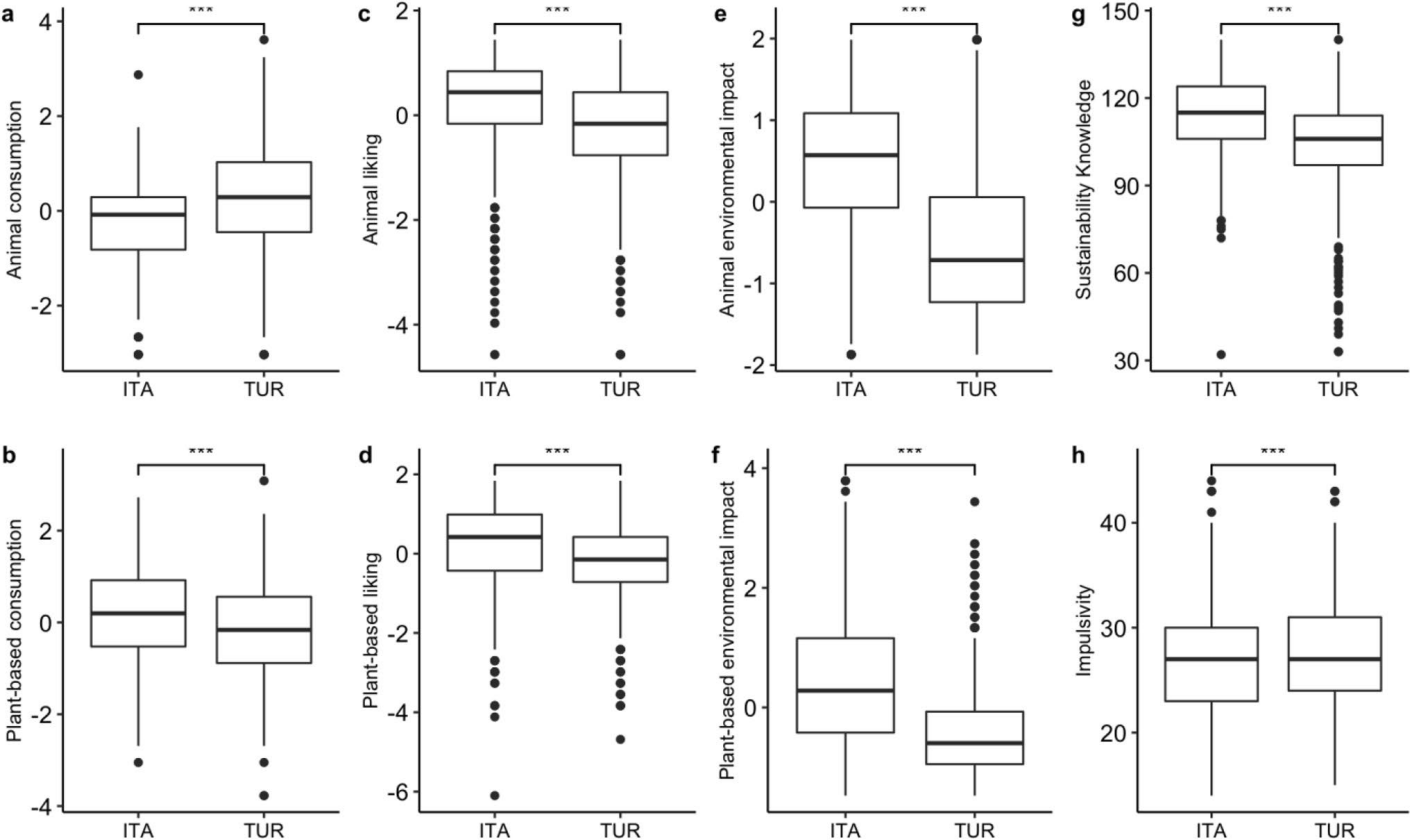
Main differences between the Italian (ITA) and the Turkish (TUR) samples. (**a**) Animal consumption, (**b**) animal liking, (**c**) animal environmental impact, (**d**) sustainability knowledge, (**e**) plant-based consumption, (**f**) plant-based liking, (**g**) plant-based environmental impact and (**h**) impulsivity traits. (**a–f**) are z-score values.

Ratings for frequency of consumption, liking, the perceived environmental impact of animal and plant-based products and the scores obtained in the impulsivity and sustainability knowledge questionnaires are reported in Fig. 1. All differences between the Italian and Turkish samples were significant. The consumption of animal products was significantly higher among Turkish respondents (t(1803.7) = 13.6, d = 0.5; see Fig. 1a) while the consumption of plant-based foods was significantly higher for Italians (t(1862.3) = 7.01, d = 0.2; see Fig. 1b). However, Italians reported significantly higher preferences for either animal and plant-based products compared to Turkish respondents (t(1875.8) = 9.53, d = 0.4 and t (1880.6) = 8.29, d = 0.3; see Fig. 1c,d). The environmental impact of both animal and plant-based products was considered on average higher by Italians (t(1818.5) = 24.61, d = 1.3 and t(1860) = 18.97, d = 0.9; see Fig. 1e,f). Finally, Italians provided higher scores in term of sustainability knowledge compared to the Turkish (t(1788.8) = 15.77, d = 2.7; see Fig. 1g), which instead turned out to be slightly more impulsive (t(1840.3) = 3.07, d = 0.3; see Fig. 1h). Within both groups, females reported higher sustainability knowledge compared to males (t(688.93) = 4.88 and t(627.64) = 4.60). Such differences were both significant (p = 0.001 and p = 0.005) and with a large effect size (d > 1). Females also expressed higher preferences for plant-based products compared to males (t(743.58) = 2.78 and t(669.96) = 3.31) in both groups. However, although such differences were significant (p = 0.005 and p < 0.001), the effect size was low for both groups (d < 0.2). The complete list of all results of Welch’s unequal variances t-test is provided in the SM (see Table S1; for a detailed analysis of the differences between the two samples for sex, generation, BMI, diet, location and educational level see from Figs. S1 to S6; see Figs. S7, S8 for correlation matrices).

**Figure 2:**
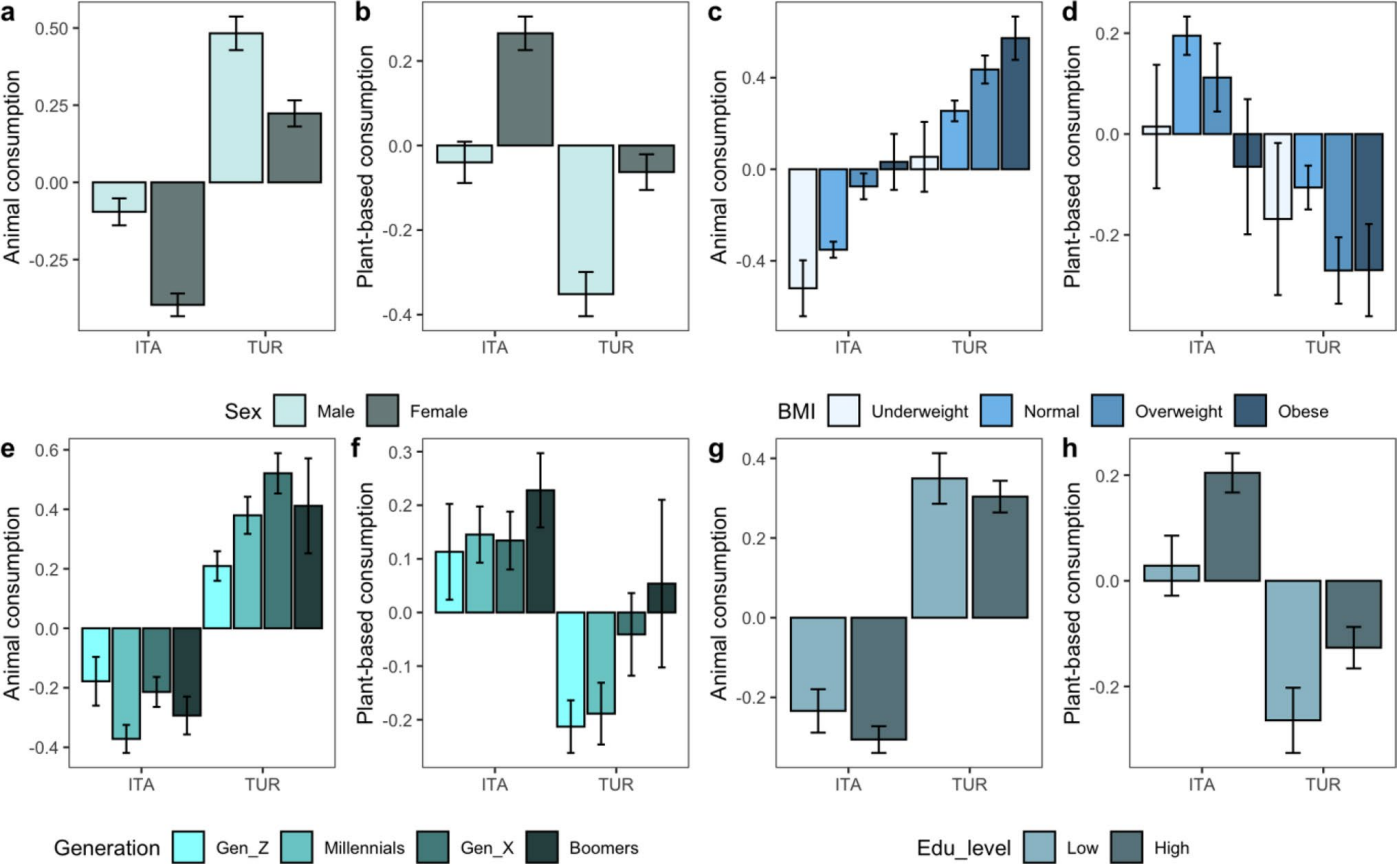
Summary of the results taking into account demographic characteristics: sex (**a,b**), BMI (**c,d**), generation (**e,f**), and education level (**g,h**) modulations on the frequency of consumption of animal- and plant-based foods among Italian (ITA) and Turkish (TUR) populations.

Results taking into account demographic characteristics and BMI modulations on the frequency of consumption of animal- and plant-based foods among the Italian and Turkish population are reported (see Fig. 2). Regardless of subject characteristics, the consumption of animal products was higher among Turkish respondents (see SM Table S2 for a summary of Welch’s unequal variances t-test results).

With respect to plant-based consumption, no significant differences were found between the two groups for underweight, overweight and obese subjects, nor for those belonging to Generation X or Boomers. Apart from these exceptions, Italian respondents had significantly higher consumption of plant-based products irrespective of subject characteristics.

Within both samples, males reported a higher frequency of consumption of animal products than females (t(830.68) = 5.28 and t(691.61) = 3.76), who instead reported consuming plant-based foods more frequently than the latter (t(808.69) = 4.85 and t(711.28) = 4.29). Such differences, although significant, had a small effect size (d = 0.2). Among Italians, Generation Z reported a significantly (p = 0.04) higher consumption of animal products compared to Millennials (t(236.71) = 2.04, p = 0.04, d = 0.2). On the contrary, within the Turkish group the consumption was significantly (p = 0.03) higher for Millennials (t(547.73) = 2.13, d = 0.1).

Across countries, plant-based consumption was significantly higher among females either in the Italian (t (808.69) = 4.84, p = 0.001, d = 0.2) and in the Turkish group (t (711.28) = 4.29, p = 0.01, d = 0.2).

In the Italian sample lower educated individuals consumed significantly (p = 0.009) less plant-based products compared to those with higher educational level (t(562.78) = 2.59, d = 0.1), while such difference was not found within the Turkish sample.

### Influence of individual variables on food consumption

For each nationality, a linear regression model was calculated to predict the frequency of animal consumption based on impulsivity and how individuals evaluated the environmental impact of animal products. Subsequently, sociodemographic factors (step 2) and eating behaviors, BMI and the interaction sustainability knowledge × sex (step 3) were added to the model. As for the animal models, for each nationality one linear regression analysis was conducted to predict the frequency of plant-based food consumption based on impulsivity and how individuals evaluated the environmental impact of plant-based products.

#### Animal consumption in the Italian sample

The linear regression for the Italian sample (see SM Table S4a) resulted in a significant model (p < 0.001) with an explained variance (adjusted R2) of 0.09; however, none of the estimated effects was significant. When adjusted for sociodemographic factors (see SM Table S5a), the model was significant (p < 0.001) and slightly increased in the explanation of the variance (adjusted R2 = 0.13). A significant negative relationship was found between being female and the consumption of food from animal origin ($\beta$ = − 0.2, SE = 0.06; p = 0.0004). A higher knowledge of sustainability was also negatively related with our dependent variable ($\beta$ = − 0.01 SE = 0.00; p < 0.001), as well as Millennials generation ($\beta$ = − 0.21 SE = 0.09; p = 0.01) and Boomers generation ($\beta$ = − 0.24 SE = 0.1; p = 0.02) compared to Generation Z(reference level). Conversely, for the variable *Location*, Suburbs ($\beta$ = 0.2 SE = 0.07; p = 0.005) and Countryside ($\beta$ = 0.2 SE = 0.07; p = 0.004) levels were significantly positively related with the consumption of animal products (compared to the reference level City). Our final model reported in Table 2 confirmed most of the previous results. However, when accounting for eating behaviors, BMI and the interaction sustainability knowledge × Sex, Obese BMI ($\beta$ = 0.3, SE = 0.13; p = 0.02) (reference level Underweight), Vegetarian ($\beta$ = 1, SE = 0.28; p = 0.0003), Flexitarian ($\beta$ = 1.3, SE = 0.28; p < 0.001) and Omnivorous diets ($\beta$ = 1.9, SE = 0.28; p < 0.001) (Vegan as reference level) were also found to be positively related with eating animal-based foods. The interaction between sex and sustainability knowledge was also significant (p = 0.018) but the estimate was very low ($\beta$ = 0.008, SE = 0.00). Contrary to the previous model, locations (i.e. Suburbs and Countryside) were not significant anymore. Overall, the model was significant (p < 0.001) with an adjusted R2 of 0.43.

**Table 2:** Coefficients, standard errors (SE) and p values for the Italian and Turkish regression models for animal-based food consumption and for plant-based food consumption.

	Animal-based consumption				Plant-based consumption			
	Italian		Turkish		Italian		Turkish	
(Intercept)	0.536	(0.36)	1.035	(0.37)	0.029	(0.41)	0.608	(0.40)
Impulsivity	0.005	(0.01)	− 0.015*	(0.01)	− 0.008	(0.01)	− 0.017 *	(0.01)
Sustainability knowledge	− 0.007 *	(0.00)	− 0.001	(0.00)	− 0.001	(0.00)	− 0.004	(0.01)
Animal environmental impact (negative)	0.106	(0.15)	0.109	(0.15)				
Plant based environmental impact (negative)					0.097	(0.13)	− 0.163	(0.19)
Impulsivity × animal environmental impact (negative)	− 0.001	(0.01)	− 0.004	(0.01)				
Impulsivity × plant based environmental impact (negative)					− 0.003	(0.01)	0.007	(0.01)
Sex (*F*)	− 0.977 *	(0.40)	0.285	(0.39)	− 0.681	(0.47)	− 0.228	(0.43)
Sex (*F*) × Sustainability knowledge	0.008 *	(0.00)	0.003	(0.00)	0.007	(0.00)	0.003	(0.00)
Education level (Low)	0.008	(0.05)	− 0.227 ***	(0.06)	0.15 *	(0.06)	0.021	(0.07)
**Generation [Z]**								
Millennials	− 0.171 *	(0.07)	0.104	(0.07)	− 0.122	(0.08)	0.019	(0.08)
X	− 0.155 *	(0.07)	0.183 *	(0.08)	− 0.087	(0.09)	0.174	(0.09)
Boomers	− 0.271 **	(0.08)	0.265	(0.15)	− 0.025	(0.10)	0.212	(0.17)
**Location [city]**								
Suburbs	0.097	(0.06)	− 0.399 **	(0.15)	0.070	(0.07)	0.068	(0.17)
Countryside	0.109	(0.06)	− 0.334 **	(0.11)	0.119	(0.07)	− 0.273 *	(0.12)
**BMI [normal]**								
Underweight	− 0.079	(0.09)	0.095	(0.11)	− 0.214 *	(0.11)	− 0.020	(0.12)
Overweight	0.204 *	(0.06)	0.018	(0.12)	0.016	(0.07)	− 0.137	(0.13)
Obese	0.291 *	(0.10)	0.150	(0.14)	0.019	(0.11)	− 0.011	(0.15)
**Diet [omnivorous]**								
Vegan	− 1.898 ***	(0.28)	− 0.912**	(0.34)	0.537	(0.33)	0.399	(0.37)
Vegetarian	− 0.891 ***	(0.19)	− 0.407	(0.24)	0.307*	(0.14)	0.132	(0.26)
Flexitarian	− 0.599 ***	(0.07)	− 0.320***	(0.09)	0.360***	(0.08)	− 0.011	(0.10)
**Dietary restrictions [none]**								
Religious beliefs	0.002	(0.49)	− 0.159 *	(0.071)	− 0.939	(0.58)	− 0.023	(0.08)
Other reasons	− 0.037	(0.08)	− 0.024	(0.09)	0.271 **	(0.09)	− 0.069	(0.10)
Allergies or Intolerances	− 0.068	(0.07)	− 0.048	(0.10)	0.057	(0.08)	0.161	(0.11)
Animal liking	0.258 ***	(0.03)	0.571 ***	(0.03)	− 0.065 *	(0.03)	− 0.091 *	(0.4)
Plant-based liking	− 0.072 **	(0.02)	− 0.04	(0.03)	0.423 ***	(0.03)	0.509 ***	(0.03)
Adjusted r-squared	0.428		0.406		0.334		0.261	
*p*	< 0.001		< 0.001		< 0.001		< 0.001	

The models are adjusted for sociodemographic characteristics, BMI, eating behaviors and the interaction between sustainability knowledge and sex.

Standard errors are in parenthesis. Reference categories are in square brackets.

Signif. codes: 0.001‘***’, 0.01‘**’, 0.05‘*’.

#### Animal consumption in the Turkish sample

The linear regression for the Turkish sample (see SM Table S4b) was also significant (p < 0.001) but explained a very small portion of the variance (adjusted R2 = 0.05). However, we found that how individuals rated the environmental impact of animal products was significantly (p = 0.03) and negatively related ($\beta$ = − 0.40, SE = 0.19) with their consumption. A small ($\beta$ = − 0.02, SE = 0.00) but significant (p = 0.018) negative value was also found for Impulsivity. When adjusted for sociodemographic characteristics (see SM Table S5b) the results of the model differed from the Italian results with the same predictors. Contrary to the latter, we found that a higher education level was significantly negatively related with eating animal products ($\beta$ = − 0.20, SE = 0.08; p = 0.01) while Generation X showed the strongest relation with consumption compared to the other generations ($\beta$ = 0.37, SE = 0.1; p = 0.0001). Unlike the Italian model, individuals living in Suburbs ($\beta$ = − 0.43, SE = 0.2; p = 0.02) or Countryside ($\beta$ = − 0.38, SE = 0.14; p = 0.006) had lower probabilities to consume animal products compared to those living in the cities. Finally, for the Turkish sample, sustainability knowledge was not a meaningful predictor (p = 0.12). In the final model (Table 2), which was significant (p < 0.001) and had an adjusted R2 of 0.41, environmental impact rating and sex were not significant (p > 0.5). Likewise, the Italian model, Omnivorous diet (but not the other diets) was strongly related to animal products consumption ($\beta$ = 0.9, SE = 0.34; p = 0.008), as well as animal liking ($\beta$ = 0.57, SE = 0.03; p < 0.001). Dietary restrictions related to religious beliefs was also found significant (p = 0.03) and strongly negatively related ($\beta$ = − 0.16, SE = 0.07).

#### Plant-based consumption in the Italian sample

The initial model (see SM Table S6a) was not significant (p = 0.09) and accounted for just a residual part of the variance (adjusted R2 = 0.004). Even when adjusted for sociodemographic factors (see SM Table S7a), albeit significant (p < 0.001), an adjusted R2 of only 0.09 was obtained. However, sex (female) was significantly related with possible higher consumption of plant-based products ($\beta$ = 0.22, SE = 0.06; p = 0.0004), as well as a high education level ($\beta$ = 0.17, SE = 0.07; p = 0.02) and sustainability knowledge ($\beta$ = 0.02, SE = 0.00; p < 0.001). In the final model reported in Table 2, however, only education level was still significant (p = 0.013) and high in magnitude ($\beta$ = 0.15, SE = 0.06). Normal BMI (p = 0.05), dietary restrictions of “other personal reasons” (p = 0.003) and liking of plant-based products (p < 0.001) were all positive and strongly associated with eating plant-based foods ($\beta$ = 0.21, SE = 0.11; $\beta$ = 0.27, SE = 0.09; and $\beta$ = 0.42, SE = 0.03 respectively). The only predictor to be significant (p = 0.05) and negatively correlated ($\beta$ = − 0.07, SE = 0.03) was animal liking. The model significantly (p < 0.001) explained the 33% of the variance.

#### Plant-based consumption in the Turkish sample

The Turkish model (see SM Table S6b), although significant (p < 0.001), was able to explain just a minimal part of the variance (adjusted r-squared = 0.03). Impulsivity was slightly ($\beta$ = − 0.03, SE = 0.01) but significantly (p < 0.001) related to a reduction in plant-based products consumption. When adjusted for sociodemographic characteristics (p < 0.001, adjusted r-squared = 0.06) (see SM Table S7b), sex (p = 0.0009) and sustainability knowledge (p = 0.003) were also found to be positively linked to the consumption of plant-based foods, but while the magnitude for sex was 0.23 (SE = 0.07) the one for sustainability knowledge was only 0.007 (SE = 0.00). In the complete model (p < 0.001, adjusted r-square = 0.26) reported in Table 2 only the effect of Impulsivity was still persistent among those reported above. The preference for plant-based food was significantly (p < 0.001) and positively linked to their consumption ($\beta$ = 0.51, SE = 0.03) while animal products liking, albeit significant (p = 0.01), was negative and mild in magnitude ($\beta$ = − 0.09, SE = 0.04). Living in the countryside was also a significant predictor (p = 0.03) and negatively related with the consumption of plant-based foods ($\beta$ = − 0.27, SE = 0.12).

## Discussion

To explore the possible role of impulsivity in moderating the effect of the perceived environmental impact of animal- and plant-based foods on food choices we employed a survey that was distributed to Italian and Turkish individuals in order to account for possible national differences. A total of 1888 responses were collected, mostly representative of young educated urban individuals.

Comparing the two populations in a cross-cultural study, we found that Turkish respondents consume significantly more animal products (poultry and bovine meat, farmed fish, eggs and cheese) than Italians, regardless of their sex, BMI, generation or educational level. Conversely, Italians reported a greater consumption of plant-based products (vegetables, pulses, tofu, nuts, fruits), performed better in the sustainability knowledge questionnaire, and considered the environmental impact of both animal- and plant-based food to be stronger. Such results can be partially explained by the apparent Italians’ greater knowledge of sustainability but also may be due to the differences between the two populations, such as the perceived climate change problem as a society, the awareness of the individuals and how proactive they are to take part in changes in their daily life, that can influence food behaviors and pro-environmental attitudes^29^.

Secondly, in both samples, females reported higher scores in the sustainability knowledge questionnaire, a stronger preference and greater consumption for plant-based products, consistent with claims in past works^72–74^. However, sex differences within groups were milder in the Turkish sample. In particular, we found that the interaction of sex and sustainability knowledge was significant for Italians but not for Turkish respondents, confirming somehow the possible role of sociocultural factors in pro-environmental behaviors^38^. A possible explanation could be that pro-environmental behavior is related to future orientation and feminism as opposite to meat consumption which was instead found to be related to masculinity, patriarchy and present orientation in previous studies^37,75–77^. Indeed, masculinity was found to be stronger in cultural groups with traditional framings of masculinity, as could be the Turkish population, compared to cultural groups that exhibit lower gender differences and where the concept of masculinity may have changed over time^42^. That is the case of Western countries, like Italy, where a changing nature of masculinity has been widely reported^78^. Indeed, Schösler et al.^77^ found a strong link between meat consumption and masculinity among Turkish men. Moreover, gender-based food stereotypes were recently confirmed among Millennials consumers in a cross-cultural comparison^79^.

Thirdly, our results show that Turkish respondents had higher in impulsivity traits, which has a strong temporal connotation to the point that the lack of futuring or forethought is considered one of its main traits^46^. Therefore, this temporal aspect of impulsivity may also influence individuals’ choices in terms of sustainability, opposing a present reward to the future of the planet. In line with the Reflective-Impulsive Model of behavior regulation^6^ participants might hold different evaluations for food items.

Moreover, our results show that for the Turkish sample Generation Zwas the one with the lowest consumption of animal products. Whereas for Italians, Millennials were the ones related to a lower consumption, although even among them Generation Z reported the highest scores in sustainability knowledge questionnaires. These results can only partly be explained by the different size of generational groups between the two populations. Indeed, this result seems to suggest that the consumption of animal products may be influenced by the perception of the climate change problem in different generations^80^. A possible explanation could be that between Italy and Turkey there might be a different timing and evolution of the society, with Generation Z in Turkey being the first generation to actively try to reduce the consumption of animal products^81^. This last result, apparently in conflict with the common view that sees younger generations acting in a more sustainable way, deserves to be further explored in future studies.

Our results also confirmed a previously found positive association between education level and the higher consumption of plant-based products in the Italian sample, but not in the Turkish respondents^26^.

Our initial hypothesis that participants with higher levels of impulsivity traits and a lower knowledge of animal-based foods environmental impact would have higher levels of animal products consumption compared to participants with lower impulsivity traits and a higher knowledge of animal-based foods environmental impact was not confirmed by the present study. However, we identified several reasons that could explain our current lack of evidence. First, it must be said that, although the samples were numerous in terms of respondents, urban highly educated individuals were over represented in both groups. Since we spread the online questionnaire mainly through social media channels and emails starting from our personal contacts, it is indeed plausible that we have reached a population that does not reflect the effective variability of the population of the two countries. In fact, in both samples, but especially in Turkish respondents, the number of highly educated individuals was significantly higher than in the actual population^82^. In the future, to ensure effective representativeness of samples, and comparisons between urban and rural inhabitants, it will be helpful to find alternative solutions for collecting responses and include more questions concerning sociodemographic characteristics (i.e. religious beliefs, political orientation^77,83,84^). A second reason may instead be related to the measure of impulsivity. Although the questionnaire was based on one of the most widely self-reported impulsivity measures, the BIS-11, the selected questions might have lacked in fully measuring impulsivity in our samples. Indeed, several studies have shown that when BIS-11 is administered in different countries, it is not uncommon for factor loadings to be low or negative, suggesting that language and culture may have a strong impact on the interpretation of each item^63^. In addition, the effectiveness of some items in properly measuring impulsivity was also questioned^59^. We should finally consider that this study was run during the exceptional period of COVID-19 pandemic, which has no comparison in recent human history. Thus, reported consumption patterns may have been different than they were before the arrival of the pandemic and the subsequent lockdowns and restrictions^85–87^. Although we did not confirm our initial hypothesis, our study provided important information on the role of impulsivity, sustainability knowledge, BMI and other sociodemographic factors in affecting the consumption of animal- and plant-based foods. More importantly, our work contributes to enrich cross-cultural literature in the food domain^36^ exploring the possible role of sociocultural and psychological factors in influencing human food behavior. In particular, it was the first study to explore a possible interaction between a psychological trait such as impulsivity with one’s knowledge of the environmental impact of food and their alimentary habits, which has been stated to play a pivotal role in contrasting climate change and environmental threats. Finally, it was conducted by a multicultural team of researchers as recently advocated by Tam and Milfont^36^.

Although our study provided several novel elements, some other limitations in addition to those listed above should be mentioned. Since we only had (biological) sex information available, it was not possible to account for gender differences, which, as mentioned above, may play an important role in food consumption. Considering the role they may have in diets^42^, future studies should take into account gender identities as well. Moreover, a few measures related to socio-economic factors were not included in this study, such as economic-status, religion, or political orientation that could have affected in part the results, in particular when comparing Italian and Turkish populations^77,83,84^. The inclusion of more predictors could have improved the fit of the model, however, this has not been done since the questionnaire consisted already of 57 questions, a high number for an online survey, and we wanted to avoid fatigue in our participants or excessive drop outs due to an excessive length of the survey (N = 221 respondents did not complete the survey as it was and such data had to be discarded). Other limitations were that, factors such as dietary habits, BMI and generation were not balanced across groups, and that it was not possible to directly address how many times per week self-reported flexitarians subjects actually consumed meat in their daily diet (although data on frequency of consumption suggest that most of them consume meat lower than three times a month).

Finally, it must be noted that Generation Z was much more represented in the Turkish sample (51.5%) than in the Italian one (14.1%), where most of the respondents belonged to Millennials (39.2%) and Generation X (29.5%). However, such distribution partially reflects the existing differences in the average age of the population between the two nations, which is 47.3 years for Italy and 31.5 years for Turkey^88^.

To overcome some of the limitations of the present study, future studies could include questions about salary, religion, or political orientation helping to understand possible sociocultural differences in sustainability-related eating behaviors and try to have age, dietary habits and BMI balanced groups.

## Conclusion

In this study we provided insights on the possible interplay between impulsivity, the perceived environmental impact of food, sustainability knowledge and food choices. Moreover, the adopted cross-cultural approach allowed us to identify several differences in the responses of participants from the two countries.

Given the importance of food choices in the environmental and health crisis, in the next few years it will be essential to change the alimentary patterns of billions of individuals towards more sustainable diets. In order to reach this purpose, it is necessary to widen the current knowledge on the relationship between psychological and sociocultural factors involved in food evaluations and understand how their interplay may influence an individual’s food choices.

## Supplementary Information

Supplementary Information.

## Data Availability

The raw data supporting the conclusions of this article will be made available by the corresponding author (r.migliavada@unisg.it), without undue reservation.
