# Supplementary material for: The modulation of sustainability knowledge and impulsivity traits on the consumption of foods of animal and plant origin in Italy and Turkey
Source: Sci Rep. 2022 Nov 21;12:20036. doi: 10.1038/s41598-022-24325-z (PMC9681846; doi:10.1038/s41598-022-24325-z)
Supplement: Supplementary file 1 — Supplementary Information. [file 41598_2022_24325_MOESM1_ESM.docx]

SUPPLEMENTARY MATERIALS

**1. Tables and Figures**

**Table S1:** Means, standard errors (SD), results of Welch’s unequal variances t-test (t) and degrees of freedom (DF), effects size (d) and p values for frequency of consumption, liking and environmental impact of animal and plant-based products and the scores obtained in the Impulsivity and Sustainability questionnaires of Italian (ITA) and Turkish (TUR) sample.

|  |  |  |  |  |  |  |  |  |  |
| --- | --- | --- | --- | --- | --- | --- | --- | --- | --- |
|  | ITA |  | TUR |  | t(DF) |  | d |  | p |
|  | M (SD) |  | M (SD) |  |  |  |  |  |  |
| Animal consumption | 2.49 (0.49) |  | 2.82 (0.55) |  | 13.61 (1803.70) |  | 0.5 |  | 0.0001*** |
| Animal liking | 5.77 (0.99) |  | 5.34 (0.96) |  | 9.53 (1875.80) |  | 0.4 |  | 0.0001*** |
| Animal environment | 4.64 (1.29) |  | 3.10 (1.42) |  | 24.61 (1818.50) |  | 1.3 |  | 0.0001*** |
| Plant-based consumption | 3.17 (0.54) |  | 2.99 (0.55) |  | 7.01 (1862.30) |  | 0.2 |  | 0.0001*** |
| Plant-based liking | 5.83 (0.71) |  | 5.57 (0.67) |  | 8.29 (1880.60) |  | 0.3 |  | 0.0001*** |
| Plant-based environment | 3.11(1.15) |  | 2.21(0.92) |  | 18.97(1860) |  | 0.9 |  | 0.0001*** |
| Sustainability | 114.5 (12.75) |  | 104.5 (14.57) |  | 15.77 (1788.80) |  | 2.7 |  | 0.0001*** |
| Impulsivity | 26.79 (4.79) |  | 27.49 (5.07) |  | 3.07 (1840.30) |  | 0.3 |  | 0.002** |

Standard errors (SD) and Degrees of freedom (DF) are in parenthesis.

Signif. codes: 0.001 ‘***’ 0.01 ‘**’ 0.05 ‘*’


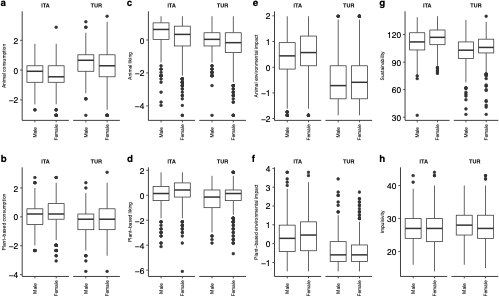


**Figure S1**: Main differences between males and females in the Italian (ITA) and the Turkish (TUR) samples. a) Animal consumption, b) Animal liking, c) Animal environmental impact, d) Sustainability knowledge, e) Plant-based consumption, f) Plant-based liking, g) Plant-based environmental impact and h) Impulsivity traits.


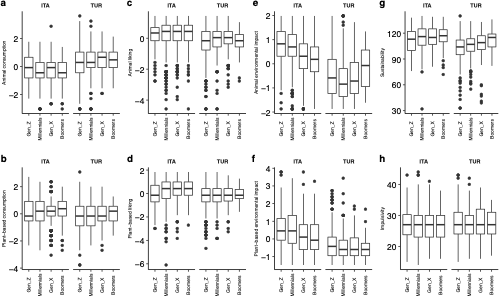


**Figure S2**: Main differences between generations in the Italian (ITA) and the Turkish (TUR) samples. a) Animal consumption, b) Animal liking, c) Animal environmental impact, d) Sustainability knowledge, e) Plant-based consumption, f) Plant-based liking, g) Plant-based environmental impact and h) Impulsivity traits.

**
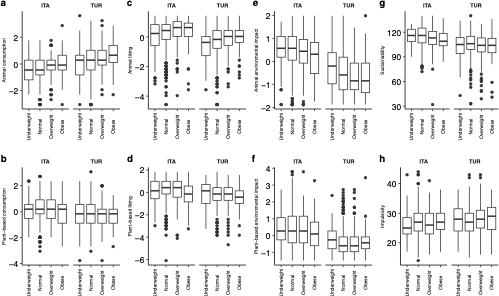
**

**Figure S3**: Main differences between BMI categories in the Italian (ITA) and the Turkish (TUR) samples. a) Animal consumption, b) Animal liking, c) Animal environmental impact, d) Sustainability knowledge, e) Plant-based consumption, f) Plant-based liking, g) Plant-based environmental impact and h) Impulsivity traits.


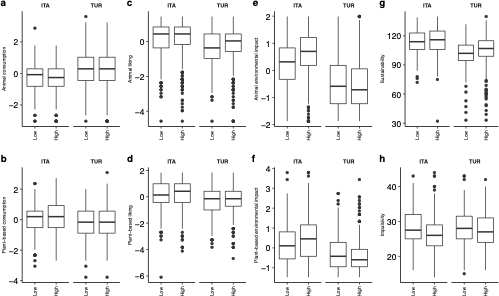


**Figure S4**: Main differences between low and high educated respondents in the Italian (ITA) and the Turkish (TUR) samples. a) Animal consumption, b) Animal liking, c) Animal environmental impact, d) Sustainability knowledge, e) Plant-based consumption, f) Plant-based liking, g) Plant-based environmental impact and h) Impulsivity traits.


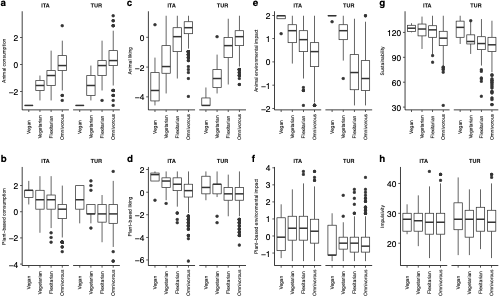


**Figure S5**: Main differences between different type of diets in the Italian (ITA) and the Turkish (TUR) samples. a) Animal consumption, b) Animal liking, c) Animal environmental impact, d) Sustainability knowledge, e) Plant-based consumption, f) Plant-based liking, g) Plant-based environmental impact and h) Impulsivity traits.


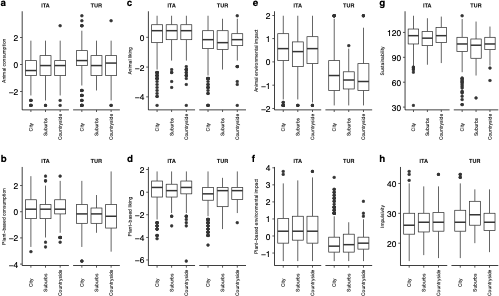


**Figure S6**: Main differences between respondents’ location of residence in the Italian (ITA) and the Turkish (TUR) samples. a) Animal consumption, b) Animal liking, c) Animal environmental impact, d) Sustainability knowledge, e) Plant-based consumption, f) Plant-based liking, g) Plant-based environmental impact and h) Impulsivity traits.

*
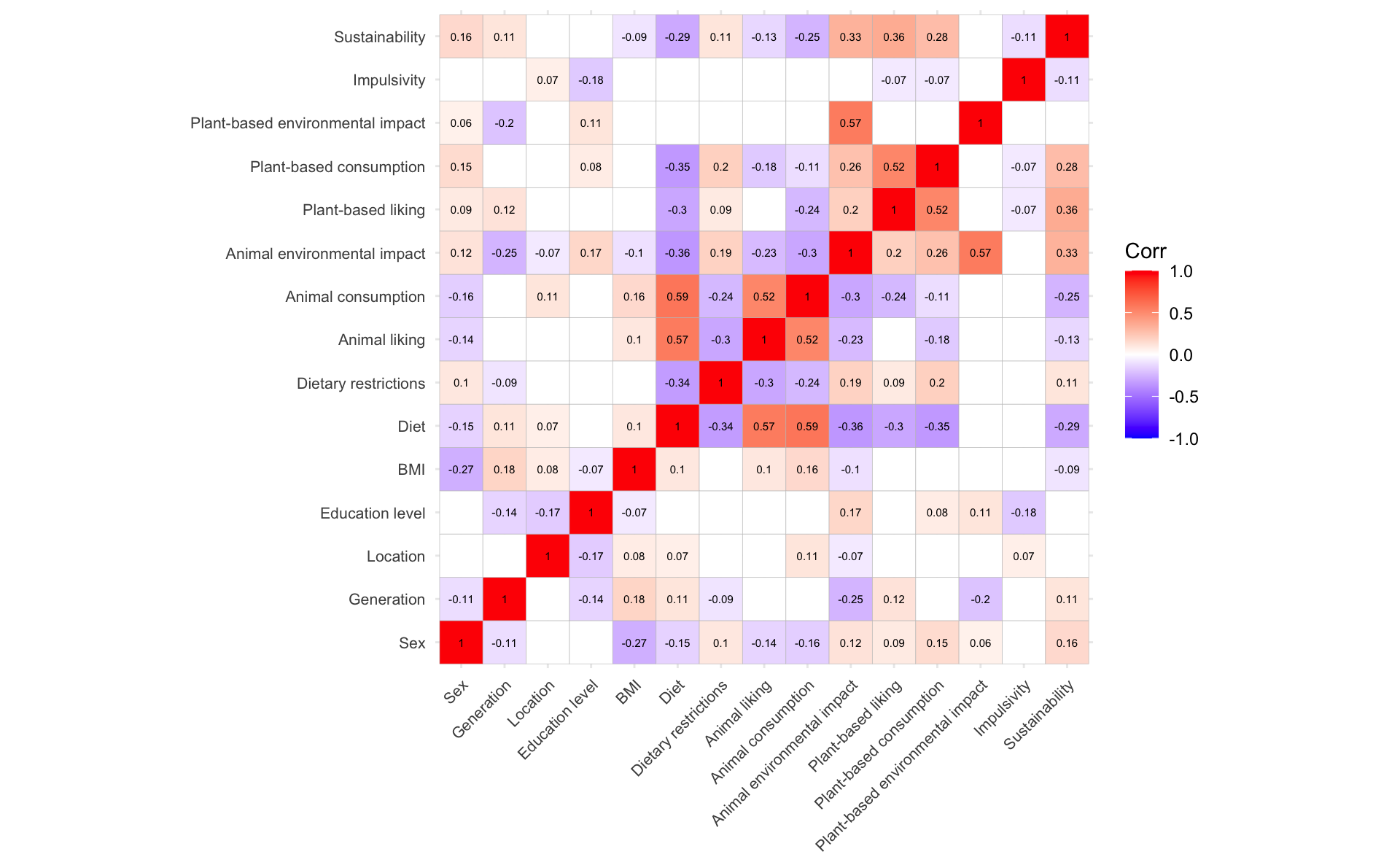
*

**Figure S7:** *Correlation matrix for the Italian sample. Significant correlations are reported, positive correlations (in red) and negative correlations (in blue).*


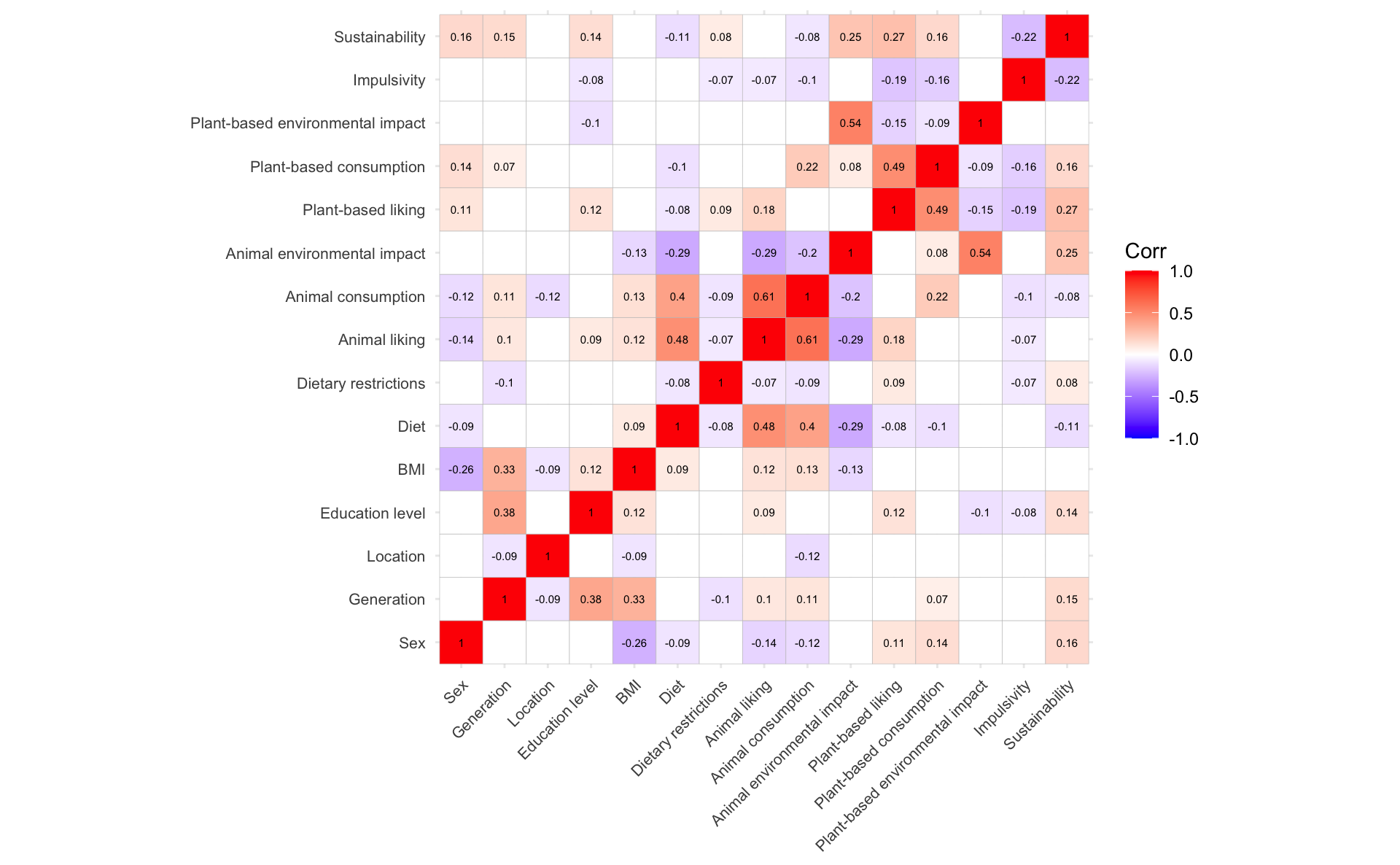


**Figure S8**: *Correlation matrix for the Turkish sample. Significant correlations are reported, positive correlations (in red) and negative correlations (in blue).*

**Table S2:** Means, standard errors (SD), results of Welch’s unequal variances t-test (t) and degrees of freedom (DF), effects size (d) and p values for Sex, BMI, Generatiion and Education level realtively to animal consumption of Italian (ITA) and Turkish (TUR) sample.

|  |  | ITA |  | TUR |  | t(DF) |  | d |  | p |
| --- | --- | --- | --- | --- | --- | --- | --- | --- | --- | --- |
|  |  | M (SD) |  | M (SD) |  |  |  |  |  |  |
| Animal consumption |  |  |  |  |  |  |  |  |  |  |
| **Sex** |  |  |  |  |  |  |  |  |  |  |
| *F* |  | 2.43 (0.50) |  | 2.77 (0.55) |  | 11.04(1152.20) |  | 0.5 |  | 0.0001*** |
| *M* |  | 2.59 (0.46) |  | 2.91 (0.53) |  | 8.29 (641.71) |  | 0.5 |  | 0.0001*** |
| **BMI** |  |  |  |  |  |  |  |  |  |  |
| *Underweight* |  | 2.36 (0.53) |  | 2.67 (0.66) |  | 2.94 (118.36) |  | 0.4 |  | 0.004** |
| *Normal* |  | 2.45 (0.49) |  | 2.78 (0.56) |  | 10.63 (1041.60) |  | 0.5 |  | 0.0001*** |
| *Overweight* |  | 2.60 (0.43) |  | 2.88 (0.49) |  | 6.13 (415.79) |  | 0.4 |  | 0.0001*** |
| *Obese* |  | 2.66 (0.51) |  | 2.95 (0.49) |  | 3.49 (120.55) |  | 0.4 |  | 0.0006*** |
| **Generation** |  |  |  |  |  |  |  |  |  |  |
| *Z* |  | 2.55 (0.53) |  | 2.76 (0.58) |  | 4.04 (249.81) |  | 0.3 |  | 0.0001*** |
| *M* |  | 2.44 (0.50) |  | 2.85 (0.54) |  | 9.61 (509.25) |  | 0.6 |  | 0.0001*** |
| *X* |  | 2.53 (0.47) |  | 2.93 (0.46) |  | 8.71 (318.35) |  | 0.6 |  | 0.0001*** |
| *Boomers* |  | 2.48 (0.45) |  | 2.87 (0.47) |  | 4.10 (38.78) |  | 0.6 |  | 0.0002*** |
| **Education level** |  |  |  |  |  |  |  |  |  |  |
| *Low* |  | 2.52 (0.51) |  | 2.83 (0.56) |  | 6.97 (539.77) |  | 0.4 |  | 0.0001*** |
| *High* |  | 2.48 (0.48) |  | 2.81 (0.54) |  | 11.73 (1261.10) |  | 0.5 |  | 0.0001*** |

Standard errors (SD) and Degrees of freedom (DF) are in parenthesis.

Signif. codes: 0.001 ‘***’ 0.01 ‘**’ 0.05 ‘*’

**Table S3:** Means, standard errors (SD), results of Welch’s unequal variances t-test (t) and degrees of freedom (DF), effects size (d) and p values for Sex, BMI, Generatiion and Education level realtively to plant-based consumption of Italian (ITA) and Turkish (TUR) sample.

|  |  | ITA |  | TUR |  | t(DF) |  | d |  | p |
| --- | --- | --- | --- | --- | --- | --- | --- | --- | --- | --- |
|  |  | M (SD) |  | M (SD) |  |  |  |  |  |  |
| Plant-based consumption |  |  |  |  |  |  |  |  |  |  |
| **Sex** |  |  |  |  |  |  |  |  |  |  |
| *F* |  | 3.24 (0.55) |  | 3.06 (0.56) |  | 5.67 (1178.80) |  | 0.2 |  | 0.0001*** |
| *M* |  | 3.07 (0.52) |  | 2.90 (0.52) |  | 4.36 (680.89) |  | 0.2 |  | 0.015* |
| **BMI** |  |  |  |  |  |  |  |  |  |  |
| *Underweight* |  | 3.10 (0.54) |  | 3.00 (0.66) |  | 0.94 (119.02) |  | 0.1 |  | 0.35 |
| *Normal* |  | 3.20 (0.55) |  | 3.03 (0.55) |  | 5.21 (1116.60) |  | 0.2 |  | 0.0002*** |
| *Overweight* |  | 3.15 (0.52) |  | 2.94 (0.54) |  | 4.0579 (413.20) |  | 0.3 |  | 0.0592 |
| *Obese* |  | 3.05 (0.57) |  | 2.94 (0.48) |  | 1.2637 (109.26) |  | 0.2 |  | 0.209 |
| **Generation** |  |  |  |  |  |  |  |  |  |  |
| *Z* |  | 3.15 (0.59) |  | 2.97 (0.58) |  | 3.20 (229.14) |  | 0.2 |  | 0.0016** |
| *M* |  | 3.17 (0.57) |  | 2.99 (0.51) |  | 4.29 (579.26) |  | 0.2 |  | 0.0211* |
| *X* |  | 3.16 (0.51) |  | 3.07 (0.53) |  | 1.86 (302.04) |  | 0.1 |  | 0.0637 |
| *Boomers* |  | 3.22 (0.50) |  | 3.12 (0.47) |  | 1.02 (41.22) |  | 0.1 |  | 0.3145 |
| **Education level** |  |  |  |  |  |  |  |  |  |  |
| *Low* |  | 3.11 (0.54) |  | 2.94 (0.56) |  | 3.49 (552.43) |  | 0.2 |  | 0.0005*** |
| *High* |  | 3.20 (0.54) |  | 3.02 (0.55) |  | 6.12 (1306.90) |  | 0.2 |  | 0.0001*** |

Standard errors (SD) and Degrees of freedom (DF) are in parenthesis.

Signif. codes: 0.001 ‘***’ 0.01 ‘**’ 0.05 ‘*’

**Table S4**: Coefficients, standard errors (SE) and p values for the Italian (a) and Turkish (b) basic regression models for animal food consumption.

|  | Animal consumption | | | | |
| --- | --- | --- | --- | --- | --- |
|  | (a) | |  | (b) | |
|  | Italian |  |  | Turkish | |
| (Intercept) | -0.308 | (0.18) |  | 0.666 *** | (0.20) |
| Impulsivity | 0.006 | (0.01) |  | -0.017 * | (0.01) |
| Animal environmental impact (negative) | 0.270 | (0.18) |  | 0.404 * | (0.19) |
| Impulsivity*Animal environmental impact (negative) | 0.002 | (0.01) |  | -0.007 | (0.01) |
| Adjusted r-squared | 0.087 |  |  | 0.047 |  |
| p | < 0.001 |  |  | < 0.001 |  |
|  |  |  |  |  |  |

Standard errors (SD) and Degrees of freedom (DF) are in parenthesis.

Signif. codes: 0.001 ‘***’ 0.01 ‘**’ 0.05 ‘*’

**Table S5**: Coefficients, standard errors (SE) and p values for the Italian (a) and Turkish (b) regression models for animal food consumption adjusted for sociodemographic characteristics.

|  | Animal consumption | | | | |
| --- | --- | --- | --- | --- | --- |
|  | (a) | |  | (b) | |
|  | Italian | |  | Turkish | |
| (Intercept) | 1.052 ** | (0.33) |  | 1.393 *** | (0.35) |
| Impulsivity | 0.004 | (0.01) |  | -0.020 ** | (0.01) |
| Animal environmental impact (negative) | 0.124 | (0.18) |  | 0.415 * | (0.18) |
| Sex (F) | -0.199 *** | (0.06) |  | -0.247 *** | (0.07) |
| **Generation [Z]** |  |  |  |  |  |
| *Millennials* | -0.212 * | (0.08) |  | 0.153 | (0.08) |
| *X* | -0.164 | (0.09) |  | 0.366 *** | (0.09) |
| *Boomers* | -0.235 * | (0.10) |  | 0.319 | (0.19) |
| **Location [City]** |  |  |  |  |  |
| Suburbs | 0.196 ** | (0.07) |  | -0.432 * | (0.19) |
| Countryside | 0.199 ** | (0.07) |  | -0.376 ** | (0.14) |
| Education level (Low) | -0.089 | (0.06) |  | 0.202 * | (0.08) |
| Sustainability Knowledge | -0.010 *** | (0.00) |  | -0.004 | (0.00) |
| Impulsivity*Animal environmental impact (negative) | 0.005 | (0.01) |  | -0.008 | (0.01) |
| Adjusted r-squared | 0.134 |  |  | 0.089 |  |
| p | < 0.001 |  |  | < 0.001 |  |
|  |  |  |  |  |  |

Standard errors (SD) and Degrees of freedom (DF) are in parenthesis.

Signif. codes: 0.001 ‘***’ 0.01 ‘**’ 0.05 ‘*’

**Figure S9:** Normal probability plot examining the distribution of residuals for the Italian (a) and Turkish (b) regression models for animal food consumption.

**
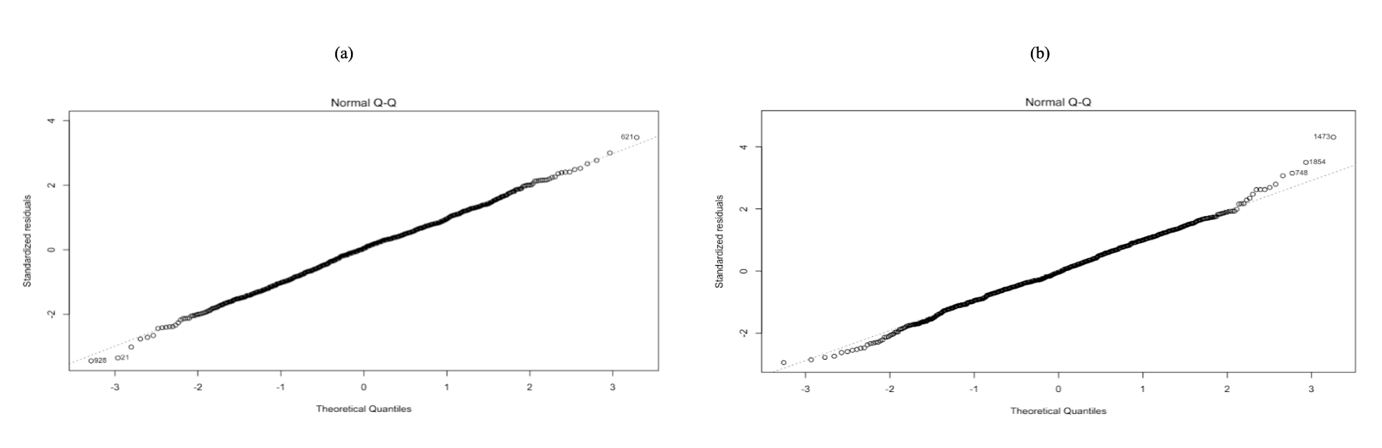
**

**Table S6**: Coefficients, standard errors (SE) and p values for the Italian (a) and Turkish (b) basic regression models for plant-based food consumption.

|  | Plant-based food consumption | | | | |
| --- | --- | --- | --- | --- | --- |
|  | (a) | |  | (b) | |
|  | Italian | |  | Turkish | |
| (Intercept) | 0.600 ** | (0.19) |  | 0.736 *** | (0.20) |
| Impulsivity | -0.017 * | (0.01) |  | -0.034 ** | (0.01) |
| Plant-based environmental impact (negative) | 0.084 | (0.16) |  | -0.142 | (0.21) |
| Impulsivity*Plant-based environmental impact (negative) | -0.004 | (0.00) |  | 0.009 | (0.01) |
| Adjusted r-squared | 0.003 |  |  | 0.030 |  |
| p | 0.093 |  |  | < 0.001 |  |
|  |  |  |  |  |  |

Standard errors (SD) and Degrees of freedom (DF) are in parenthesis.

Signif. codes: 0.001 ‘***’ 0.01 ‘**’ 0.05 ‘*’

**Table S7**: Coefficients, standard errors (SE) and p values for the Italian (a) and Turkish (b) regression models for plant-based food consumption adjusted for sociodemographic characteristics.

|  | Plant-based food consumption | | | | |
| --- | --- | --- | --- | --- | --- |
|  | (a) | |  | (b) | |
|  | Italian | |  | Turkish | |
| (Intercept) | -2160 *** | (0.35) |  | 0.340 *** | (0.34) |
| Impulsivity | -0.008 | (0.01) |  | -0.028 ** | (0.01) |
| Plant-based environmental impact (negative) | 0.109 | (0.16) |  | -0.160 | (0.21) |
| Sex (F) | 0.224 *** | (0.06) |  | 0.228 | (0.07) |
| **Generation [Z]** |  |  |  |  |  |
| *Millennials* | -0.071 | (0.09) |  | -0.019 | (0.08) |
| *X* | -0.042 | (0.10) |  | 0.093 | (0.09) |
| *Boomers* | 0.052 | (0.11) |  | 0.150 | (0.19) |
| **Location [City]** |  |  |  |  |  |
| *Suburbs* | 0.024 | (0.08) |  | 0.033 | (0.19) |
| *Countryside* | 0.124 | (0.08) |  | -0.227 | (0.14) |
| Education level (Low) | -0.169 * | (0.07) |  | -0.060 | (0.08) |
| Sustainability Knowledge | 0.020 *** | (0.00) |  | 0.007 | (0.00) |
| Impulsivity*Plant-based environmental impact (negative) | -0.004 | (0.00) |  | 0.009 | (0.01) |
| Adjusted r-squared | 0.003 |  |  | 0.056 |  |
| p | 0.093 |  |  | < 0.001 |  |
|  |  |  |  |  |  |

Standard errors (SD) and Degrees of freedom (DF) are in parenthesis.

Signif. codes: 0.001 ‘***’ 0.01 ‘**’ 0.05 ‘*’

**Figure S10:** Normal probability plot examining the distribution of residuals for the Italian (a) and Turkish (b) regression models for plant-based food consumption.


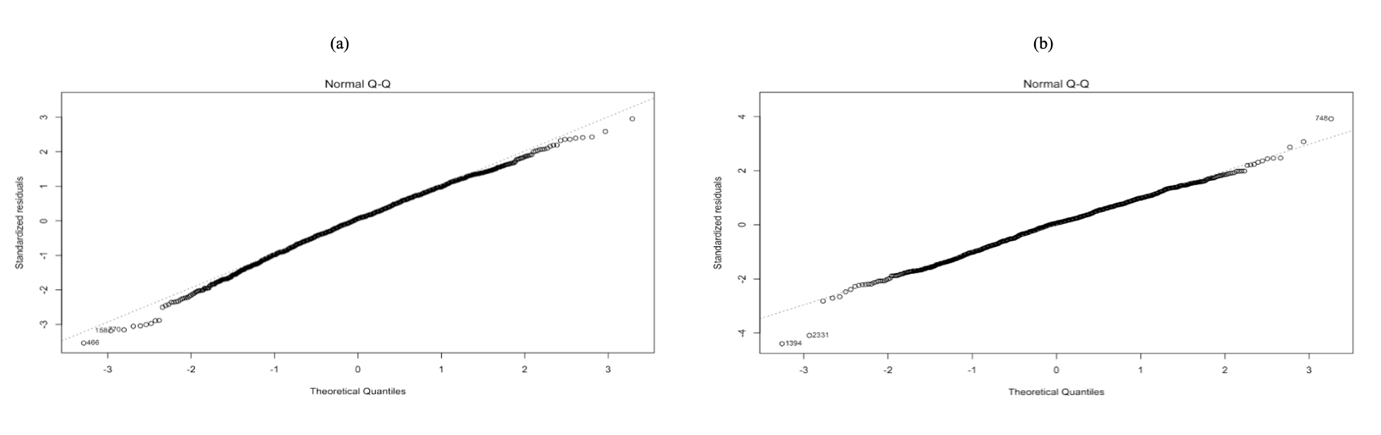


**Figure S11:** Frequency of food consumption in Italy and Turkey

**
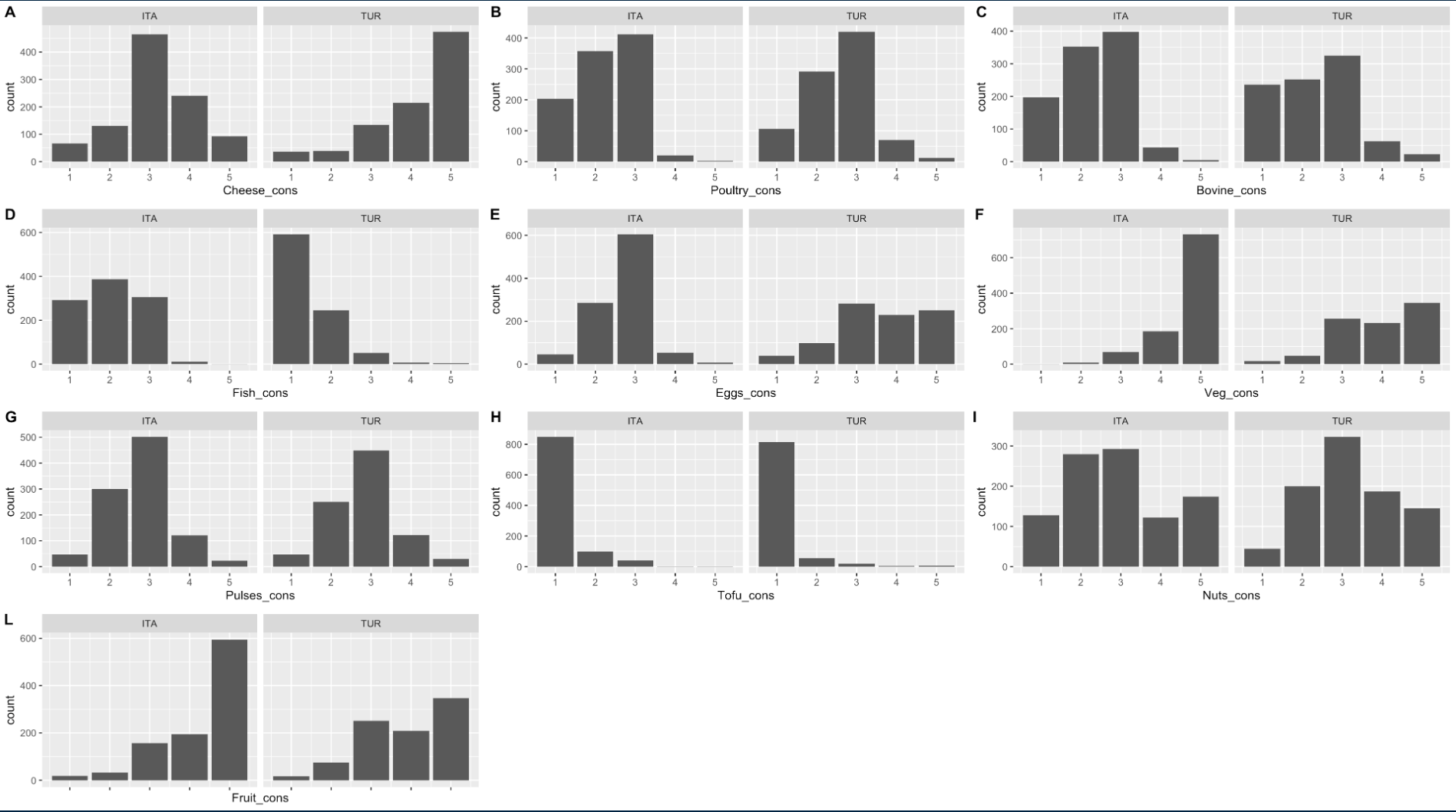
**

(Where 1 is less than once a month and 5 is at least once a day)

**2. Questionnaire**

Below the complete online survey in the English version (available Italian and Turkish versions).

**Start of Block: LANGUAGE**

SELECT YOUR LANGUAGE - SELEZIONA LA TUA LINGUA - DILINIZI SEÇINIZ

**End of Block: LANGUAGE**

**Start of Block: INTRODUCTION**

Thank you for agreeing to participate in our study. The study is conducted by the University of Gastronomic Sciences in Pollenzo (Italy) and takes about 15 minutes to be completed.
You will be asked to answer some questions about your alimentary habits. There are no correct or wrong answers, just answer what you spontaneously think.
Participation in the study is completely voluntary. You may withdraw from the study without giving reasons at any time.
Your responses will be stored under a randomly allocated participant number. Responses will therefore remain completely anonymous.
The anonymous data will be combined with others for analysis. This fully anonymous data may be deposited online in scientific research databases or submitted alongside a manuscript to a scientific journal. Once such material has been made available online we cannot rule out further distribution by others, as this is beyond the responsibility or influence of University of Gastronomic Sciences. For this reason, we will only ever upload or make available fully anonymous data.
 

According to the Regulation (EU) 2016/679 approved  by the European Parliament and Council and according to the Italian law no. 101/18 of 10th August 2018 and its amendments published in the Official Journal no. 205 of   04th September 2018 (protection of persons and other subjects regarding the processing of personal data) the processing of the information provided by you will be deployed in accordance with principles of correctness, lawfulness and protection of your privacy and your rights.
 
I have noted the above conditions and I agree to participate anonymously in the study:

- **I agree**
- **I do not agree**

**End of Block: INTRODUCTION**

**Start of Block: DEMOGRAPHIC**

Sex:

- Male
- Female

| 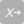 |
| --- |

Age:

▼ ... > 90

| 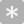 |
| --- |

Height (cm):

________________________________________________________________

| 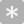 |
| --- |

Weight (kg):

________________________________________________________________

Nationality:

- Afghanistan
- …
- Zimbabwe

Have you lived in your country of residence for at least 10 years?

- Yes
- No

Education:

- No schooling completed/Primary school
- Middle school
- Some High School, no diploma
- High school graduate
- Bachelor's Degree
- Master's Degree
- Ph.D.

Diet:

- Vegan
- Vegetarian
- Flexitarian (primarily vegetarian diet but occasionally includes meat or fish)
- Omnivorous

Do you have dietary restrictions for one of the following reasons?

- No, I don't
- Yes, Allergies, and/or Intolerances
- Yes, Religious beliefs
- Yes, Other personal reasons

Where do you live?

- City
- Suburbs
- Countryside

**End of Block: DEMOGRAPHIC**

**Start of Block: INTRODUCTION ITEMS**

In the following section you will be introduced to 10 different foods and asked to answer some questions. Answer quickly and without thinking too much as there are no right or wrong answers.

**End of Block: INTRODUCTION ITEMS**

**Start of Block: ITEM 1**

POULTRY MEAT

How much do you like the indicated food product?

- Dislike very much
- Dislike moderately
- Dislike slightly
- Neither like nor dislike
- Like slightly
- Like moderately
- Like very much

How often do you consume the indicated food product?

- Less than once a month
- 1-3 times a month
- 1-3 times a week
- 4-6 times a week
- At least once a day

Do you think that the consumption of the indicated food product can have a negative impact on the planet or cause in some way any damage?

- Definitely NO
- NO
- Probably NO
- I don’t know
- Probably YES
- YES
- Definitely YES

**End of Block: ITEM 1**

**Start of Block: ITEM 2**

BOVINE MEAT

How much do you like the indicated food product?

- Dislike very much
- Dislike moderately
- Dislike slightly
- Neither like nor dislike
- Like slightly
- Like moderately
- Like very much

How often do you consume the indicated food product?

- Less than once a month
- 1-3 times a month
- 1-3 times a week
- 4-6 times a week
- At least once a day

Do you think that the consumption of the indicated food product can have a negative impact on the planet or cause in some way any damage?

- Definitely NO
- NO
- Probably NO
- I don’t know
- Probably YES
- YES
- Definitely YES

**End of Block: ITEM 2**

**Start of Block: ITEM 3**

CHEESE

How much do you like the indicated food product?

- Dislike very much
- Dislike moderately
- Dislike slightly
- Neither like nor dislike
- Like slightly
- Like moderately
- Like very much

How often do you consume the indicated food product?

- Less than once a month
- 1-3 times a month
- 1-3 times a week
- 4-6 times a week
- At least once a day

Do you think that the consumption of the indicated food product can have a negative impact on the planet or cause in some way any damage?

- Definitely NO
- NO
- Probably NO
- I don’t know
- Probably YES
- YES
- Definitely YES

**End of Block: ITEM 3**

**Start of Block: ITEM 4**

FARMED FISH

How much do you like the indicated food product?

- Dislike very much
- Dislike moderately
- Dislike slightly
- Neither like nor dislike
- Like slightly
- Like moderately
- Like very much

How often do you consume the indicated food product?

- Less than one a month
- 1-3 times a month
- 1-3 times a week
- 4-6 times a week
- At least once a day

Do you think that the consumption of the indicated food product can have a negative impact on the planet or cause in some way any damage?

- Definitely NO
- NO
- Probably NO
- I don’t know
- Probably YES
- YES
- Definitely YES

**End of Block: ITEM 4**

**Start of Block: ITEM 5**

EGGS

How much do you like the indicated food product?

- Dislike very much
- Dislike moderately
- Dislike slightly
- Neither like nor dislike
- Like slightly
- Like moderately
- Like very much

How often do you consume the indicated food product?

- Less than once a month
- 1-3 times a month
- 1-3 times a week
- 4-6 times a week
- At least once a day

Do you think that the consumption of the indicated food product can have a negative impact on the planet or cause in some way any damage?

- Definitely NO
- NO
- Probably NO
- I don’t know
- Probably YES
- YES
- Definitely YES

**End of Block: ITEM 5**

**Start of Block: ITEM 6**

VEGETABLES

How much do you like the indicated food product?

- Dislike very much
- Dislike moderately
- Dislike slightly
- Neither like nor dislike
- Like slightly
- Like moderately
- Like very much

How often do you consume the indicated food product?

- Less than once a month
- 1-3 times a month
- 1-3 times a week
- 4-6 times a week
- At least once a day

Do you think that the consumption of the indicated food product can have a negative impact on the planet or cause in some way any damage?

- Definitely NO
- NO
- Probably NO
- I don’t know
- Probably YES
- YES
- Definitely YES

**End of Block: ITEM 6**

**Start of Block: ITEM 7**

PULSES

How much do you like the indicated food product?

- Dislike very much
- Dislike moderately
- Dislike slightly
- Neither like nor dislike
- Like slightly
- Like moderately
- Like very much

How often do you consume the indicated food product?

- Less than once a month
- 1-3 times a month
- 1-3 times a week
- 4-6 times a week
- At least once a day

Do you think that the consumption of the indicated food product can have a negative impact on the planet or cause in some way any damage?

- Definitely NO
- NO
- Probably NO
- I don’t know
- Probably YES
- YES
- Definitely YES

**End of Block: ITEM 7**

**Start of Block: ITEM 8**

TOFU

How much do you like the indicated food product?

- Dislike very much
- Dislike moderately
- Dislike slightly
- Neither like nor dislike
- Like slightly
- Like moderately
- Like very much

How often do you consume the indicated food product?

- Less than once a month
- 1-3 times a month
- 1-3 times a week
- 4-6 times a week
- At least once a day

Do you think that the consumption of the indicated food product can have a negative impact on the planet or cause in some way any damage?

- Definitely NO
- NO
- Probably NO
- I don’t know
- Probably YES
- YES
- Definitely YES

**End of Block: ITEM 8**

**Start of Block: ITEM 9**

NUTS (e.g. almonds, cashew nuts, hazelnuts, pistachios, walnuts etc.)

How much do you like the indicated food product?

- Dislike very much
- Dislike moderately
- Dislike slightly
- Neither like nor dislike
- Like slightly
- Like moderately
- Like very much

How often do you consume the indicated food product?

- Less than once a month
- 1-3 times a month
- 1-3 times a week
- 4-6 times a week
- At least once a day

Do you think that the consumption of the indicated food product can have a negative impact on the planet or cause in some way any damage?

- Definitely NO
- NO
- Probably NO
- I don’t know
- Probably YES
- YES
- Definitely YES

**End of Block: ITEM 9**

**Start of Block: ITEM 10**

FRUITS

How much do you like the indicated food product?

- Dislike very much
- Dislike moderately
- Dislike slightly
- Neither like nor dislike
- Like slightly
- Like moderately
- Like very much

How often do you consume the indicated food product?

- Less than once a month
- 1-3 times a month
- 1-3 times a week
- 4-6 times a week
- At least once a day

Do you think that the consumption of the indicated food product can have a negative impact on the planet or cause in some way any damage?

- Definitely NO
- NO
- Probably NO
- I don’t know
- Probably YES
- YES
- Definitely YES

**End of Block: ITEM 10**

**Start of Block: IMPULSIVITY**

People differ in the ways they act and think in different situations. This is a test to measure some of the ways in which you act and think. Read each statement and select the appropriate answer. Do not spend too much time on any statement. Answer quickly and honestly.

|  | Rarely / Never | Occasionally | Often | Almost always / Always |
| --- | --- | --- | --- | --- |
| I plan tasks carefully. |  |  |  |  |
| I plan trips well ahead of time. |  |  |  |  |
| I am self controlled. |  |  |  |  |
| I save regularly. |  |  |  |  |
| I am a careful thinker |  |  |  |  |
| I plan for job security. |  |  |  |  |
| I say things without thinking. |  |  |  |  |
| I like to think about complex problems. |  |  |  |  |
| I change jobs. |  |  |  |  |
| I get easily bored when solving thought problems. |  |  |  |  |
| I change residences. |  |  |  |  |
| I can only think about one thing at a time. |  |  |  |  |
| I am more interested in the present than the future. |  |  |  |  |
| I like puzzles. |  |  |  |  |
| I am future oriented. |  |  |  |  |

**End of Block: IMPULSIVITY**

**Start of Block: SUSTAINABILITY KNOWLEDGE**

The survey that you are going to take is about food and the environment. Please indicate your agreement with the following statements.

|  | Strongly disagree | Disagree | Disagree somewhat | Neither agree nor disagree | Agree somewhat | Agree | Strongly agree |
| --- | --- | --- | --- | --- | --- | --- | --- |
| A vegetarian diet can reduce greenhouse gas emissions. |  |  |  |  |  |  |  |
| Assurance of animal welfare in food production is important to me. |  |  |  |  |  |  |  |
| Consuming products made from environmentally friendly grains is more expensive than consuming conventional products. |  |  |  |  |  |  |  |
| Consuming seasonal vegetables is environmentally friendly. |  |  |  |  |  |  |  |
| Conventional and highly automated farming leads to higher quality products. |  |  |  |  |  |  |  |
| Intensive agriculture leads to reduced biodiversity which I find unacceptable. |  |  |  |  |  |  |  |
| Food/Gastronomic/Agricultural tourism can help the development and sustainability of small local farmers. |  |  |  |  |  |  |  |
| I am willing to pay a slightly higher price for local foods. |  |  |  |  |  |  |  |
| The less food packaging the more sustainable the food. |  |  |  |  |  |  |  |
| I pay attention to environmental information on food labels. |  |  |  |  |  |  |  |
| I prefer buying food from local or nearby markets/producers. |  |  |  |  |  |  |  |
| I will avoid producers and products that I know have a high impact on the environment. |  |  |  |  |  |  |  |
| If the price is reasonable, I will buy foods produced using sustainable strategies. |  |  |  |  |  |  |  |
| Reducing land use, fresh water consumption, and fossil fuels used in food production should be an important goal of food producers. |  |  |  |  |  |  |  |
| Small farmers are essential to guarantee farming sustainability in the world. |  |  |  |  |  |  |  |
| Social aspects of food production (for example, fair trade, social right of workers) are important to me. |  |  |  |  |  |  |  |
| Sustainable agriculture must be concerned with ensuring the economic viability of the farm and the farmer. |  |  |  |  |  |  |  |
| The price I pay for organic or more sustainable foods is worth it. |  |  |  |  |  |  |  |
| The volume of water needed to grow 1 lb of tomatoes is approximately the same as the amount needed to grow 1 lb of wheat. |  |  |  |  |  |  |  |
| World food production cannot be maintained through local products; intensive agriculture is needed. |  |  |  |  |  |  |  |
| Even if the price of organic products is slightly higher than that of conventional products, I will buy the organic products. |  |  |  |  |  |  |  |
| When I choose local foods, I reduce transporting and packaging costs. |  |  |  |  |  |  |  |

**End of Block: SUSTAINABILITY KNOWLEDGE**
